# Effects of Transport Conditions on Behavioural and Physiological Responses of Horses

**DOI:** 10.3390/ani10010160

**Published:** 2020-01-17

**Authors:** Barbara Padalino, Sharanne L Raidal

**Affiliations:** 1 Department of Agricultural and Food Sciences, University of Bologna, Viale Fanin 44, 40127 Bologna, Italy; 2 School of Animal and Veterinary Sciences, Charles Stuart University, Wagga, NSW 2650, Australia; sraidal@csu.edu.au

**Keywords:** travelling, welfare, space, position, behaviour, stomach ulcers, equine

## Abstract

**Simple Summary:**

The aim of this study was to document the effects of 12 hours’ confinement in comparison with 12 h of transportation in single and wide bays, and in backward and forward positioning, on horse behavioural, physiological, laboratory and gastroscopy parameters. Behaviours relating to stress and balance occurred more frequently during transport than during confinement, and transport in a rear-facing position and in a wider bay size were associated with reduced balance-related behaviours. An increased frequency of balance behaviours, in particular loss of balance, and transport-related increases in heart rate and rectal temperature were associated with gastric ulceration after transportation. While effects of bay size and direction of travel on stress behaviours were less clear and require further study, this study suggests that adequate space and rear-facing positioning facilitates better balance and may enhance the health and welfare of transported horses. Behavioural observations, heart rate and monitoring of rectal temperature are useful to identify horses at risk for development of transport-related diseases.

**Abstract:**

The regulations for minimal space and direction of travel for land transport in horses vary worldwide and there is currently no definitive guidance to promote equine health and welfare. This study evaluated the effects of bay size and direction of travel (forwards/backwards) in horses by comparing the behavioural, physiological, laboratory and gastroscopy parameters between transported and confined horses. A total of twenty-six mares took part in the study; 12 horses were confined for 12 h, and all mares underwent 12 hours’ transportation, travelling in single (n = 18) or wide bays (n = 8), and forward (n = 10) or rear (n = 16) facing. Behaviour was recorded during confinement/transportation and analysed using a behaviour sampling ethogram. Clinical examination, blood samples and gastroscopy were conducted before and after confinement/transportation. The frequency of behaviours relating to stress and balance increased during transport, and horses transported in a rear-facing position and in a wider bay size showed fewer balance-related behaviours. Balance behaviours, particularly loss of balance, were positively associated with the severity of gastric ulceration after transportation and elevated muscle enzymes, while increased stress behaviours correlated with decreased gastrointestinal sounds. Heart rate and rectal temperature after transportation were positively associated with balance and stress behaviours, and with squamous gastric ulcer scores. Transportation was associated with expected increases in cortisol and muscle enzymes, but positioning and space allowance had minimal effects on these analytes. Findings suggest that transportation in a rear-facing position and in wider bays might reduce the impact of transport on horse health and welfare, and monitoring behaviour in transit and physiological measurements after transportation should be recommended. Behavioural and physiological parameters were more sensitive than haematological, biochemical or endocrine analytes to identify horses suffering from transport stress.

## 1. Introduction

Transportation has been identified as a stressor for horses, and has been associated with several adverse outcomes including injury, respiratory and gastrointestinal disease [1,2,3,4,5,6]. We have recently shown that 12 hours’ transportation is associated with ulceration of the gastric squamous mucosa in fasted horses, associated with increased pH of gastric content, and possibly with decreased gastrointestinal motility in horses fed 1 h and 6 h prior to transportation [7]. Animal management during transportation may influence disease outcomes [8,9,10], and international regulations on land transportation of live animals have been updated based on recent publications to safeguard the welfare of transported animals. However, there is still no agreement in mandatory requirements between countries and evidence in support of some recommendations is limited.

The adverse effects of transportation may be affected by confinement, isolation, direction of travel, and the size of the compartment in which the horse is transported [11]. Several studies have been performed in order to determine the effects of direction of travel on a horses’ ability to maintain balance during transport of different duration (from 17 min to 3 h) [12,13,14,15], but results have often been conflicting due to differences in trailer design, journey duration, and lack of simultaneous comparisons. Similarly, there is no agreement on the space allowance needed in transit [16,17], and variable minimal space allowances are reported in current transport regulations of different countries [18,19,20]. It has been reported that the most commonly observed body posture in horses during transportation involves standing with the front and hind limbs apart and the forelegs stretched forward [21] a postural adaptation likely to help the horse to retain its balance. However, to assume this position, horses need sufficient space between their body and the vehicle partitions or other horses. Similarly, beneficial effects associated with lowering of the head below the height of the withers have been well characterised during journeys longer than 8 h [5,22], and this posture also requires a space allowance greater than currently available in many transport vehicles.

This study documented the effects of 12 hours’ confinement in comparison with 12 h of transportation in single or wide bays, and in backward or forward positioning, on behavioural, physiological, laboratory and gastroscopy parameters. It was hypothesized that behaviours relating to stress and balance and physiological, laboratory and gastroscopy parameters would be increased in transported horses relative to those observed in confined horses, and that transportation in a rear-facing position and a wider bay size would attenuate such changes. We also hypothesized that behaviour would predict the severity of gastric ulceration (increased squamous and glandular ulcer scores), and that the frequency of behaviours related to stress would be correlated with increases in cortisol, muscle enzymes, rectal temperature and heart rate in transported horses.

## 2. Materials and Methods

### 2.1. Animals

Twenty-six light breed mares, aged from 4 to 20 years (mean 9.9 years) with mean body weight of 518.8 kg (range 416 to 658 kg) were recruited for this study. All horses were CSU teaching or research horses, and had been resident on site for four or more weeks. Prior transport history was unknown for each horse, although all had been transported on at least one prior occasion without adverse reaction. All were well accustomed to handling, were healthy on veterinary evaluation and judged fit for transportation [23]. Except during transportation and confinement, horses were kept on pasture, fed alfalfa hay twice a day (08:00 h; 18:00 h), and had water ad libitum. The diet was calculated individually to meet maintenance requirements (1 to 1.5% body weight). Feeding was manipulated for confinement and transportation as described below. All experimental procedures were approved by the Animal Care and Ethics Committee, Charles Sturt University, NSW, Australia (authorisation n A17011).

### 2.2. Experimental Protocol

The experimental protocol has been described previously [7]. Briefly, the study was conducted in two parts. Part 1 was conducted to assess the effect of overnight confinement (18:00 h to 06:00 h), without feeding, in 12 mares. Part 2 was conducted to determine the effect of overnight (18:00 h to 06:00 h) transportation in 26 mares, including the twelve mares used in study Part 1.

Part 1: Mares (n = 12) were confined in reproductive stocks (148 × 71 cm, height of front gate 112 cm) as two groups, each of six horses, on consecutive nights. Horses were tied loosely with a cord of approximately 60 cm to the front of the stocks, in the same manner as they would be restrained during transportation. Horses were fed alfalfa hay between 06:00 h and 07:00 h on the morning of confinement. Water was withheld from 12:00 h. Each horse underwent veterinary clinical examination and venous blood was collected for haematology, serum biochemistry and blood gas analysis at 14:00 h, prior to confinement (T0). Intestinal borborygmi were graded subjectively by auscultation of four abdominal quadrants (upper left, lower left, upper right, lower right) as 0 (no intestinal sounds auscultated in 60 s), 1 (decreased activity), 2 (normal activity, 2 or 3 discrete rumbling or gurgling noises in 30s) or 3 (increased activity) for each quadrant. These results were summed to give a gastrointestinal (GI) activity score, as previously described [24]. Horses were sedated (200 mg xylazine and 10 mg acetylpromazine, or 10 mg detomidine and 5 mg butorphanol by intravenous injection) between 15:00 h and 17:00 h for gastroscopy. During confinement (18:00 h to 06:00 h), horses were monitored continuously by one author (BP) placed in the adjoining room thought a glass wall, their behaviour was video recorded continuously, but were not offered food or water. Clinical examination, venous blood collection and gastroscopy were repeated at the end of confinement, at 06:00 h the following day (T1).

**Figure 1:**
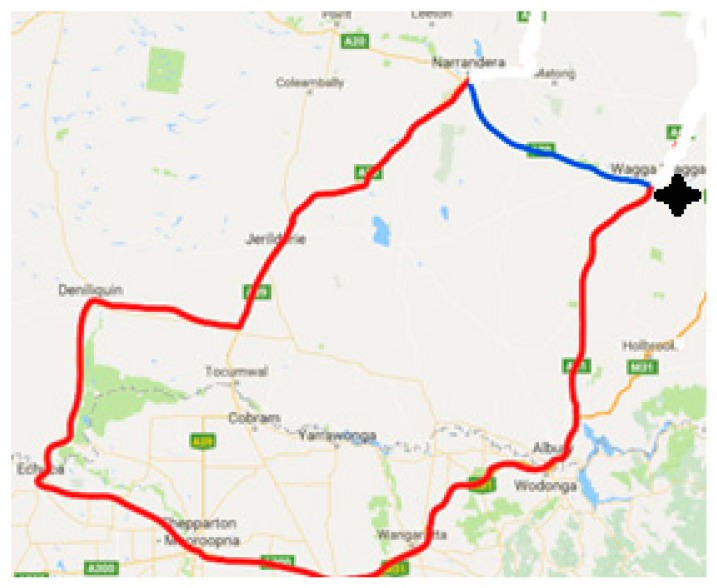
Road transport route travelled by all horses in Part 2 of the study, departing from Wagga Wagga at 18:00 h and returning after 12 h. The route covered a distance of 750 km and proceding in a clockwise direction.

Part 2: Effects of transportation were assessed in 26 horses travelled as two consignments, each of 13 horses, on consecutive nights (Trip 1, Trip 2). Both trips were completed 14 days after study Part 1, over an identical route (Figure 1) covering approximately 880 km, with the same driver and vehicle, departing at 6 pm (18:00 h) and returning at 6am the following morning (06:00 h).

**Figure 2:**
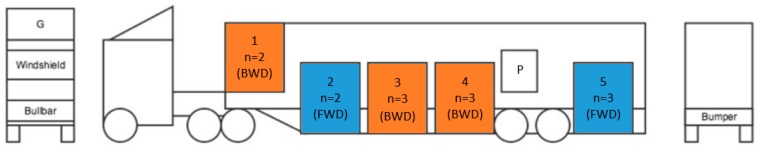
Truck configuration—the truck was sectioned into 5 compartments; blue bays are forward facing, orange bays are rear facing (BWD). Partitioning into single and wide bays is indicated as wide bays accommodated two horses (n = 2), and single bays accommodated three horses (n = 3) in each compartment. A personnel compartment (*p*) with seating for three people was located over the trailer’s rear wheels, with continuous access to the horses in compartments 4 and 5. Legend: FWD: forward facing direction; BWD: backward facing direction

The transport vehicle was a 15-horse trailer attached to a prime mover (LF290 18T, DAF Trucks, Bayswater, Victoria, Australia). The trailer was split into three sections (Figure 2). The first section was a raised platform above the prime mover’s drive axle, with two wide bays (190 × 100 cm, each) where horses travelled backwards (i.e., with the rear end in the direction of travel). The second section was a dropped platform in front of the trailer’s axle, where there were three compartments. In the first compartment two horses travelled facing forward in wide bays (190 × 112 cm, each), in the second and third compartment 6 horses travelled backwards in standard single bays (190 × 76 cm, each). The third section of the trailer was after the trailer’s axle, and three horses travelled facing forward in single bays (190 × 76 cm). On each journey, six of the 12 horses used in Part 1 of the study travelled in the fourth and fifth compartment in single bays, located at the back of the truck.

The twelve horses used for Part 1 were fed between 06:00 h and 07:00 h on the morning of transportation, prior to confinement. Water was removed at 12:00 h, and horses underwent gastroscopic examination between 16:00 h and 18:00 h. Gastroscopy was performed on the remaining 14 horses on the day prior to transportation, approximately 24h prior to departure (T-1), and horses were fed 1h prior to transportation (trip 1, n = 7) or 6 h prior to transportation (trip 2, n = 7), as previously described [7]. Gastroscopy results from T-1 were pooled with results from T0 for analysis of all pre-transportation observations.

Clinical examination and venous blood collection were performed as in Part 1, at 14:00 h, approximately 4h before departure. All horses loaded easily, without loading behavioural problems, which could have suggested evidence of prior adverse experience of transportation. Horses were monitored continuously during transit by video camera, with researchers travelling in the vehicle. Clinical examination, venous blood collection and gastroscopy were repeated for all horses at the end of transportation (T1).

### 2.3. Behavioural Parameters

Horses were recorded during confinement by a security camera system (TechView DVR Kit, Model Number QV-3034, Petaling Jaya, Selangor, Malaysia) placed in front of each stock. During transport, a camera was placed in each compartment of the trailer, pointing toward the horses’ heads, enabling each horse’s behaviour to be recorded continuously during the journey. A behaviour sampling ethogram (Table 1) was developed based on those used previously to study behaviour during transportation [5,25]. De-identified videos were analysed by an experienced ethologist (BP) using a time window defined as the first 20 min of each hour during transportation or confinement. Behavioural assessment was performed independently of clinical, gastroscopic and laboratory findings for each horse, blinded to the position inside the truck (rear or forward facing) but, unavoidably, not blinded to the situation (confinement or transportation) or available space (single or wide bays).

### 2.4. Gastroscopy

Squamous and glandular gastric ulceration were scored separately as previously described [31]. Briefly, a validated equine scoring system [32] was used in real time and on review of de-identified video-recordings to give separate scores for the squamous mucosa of the greater curvature, lesser curvature and fundus, which were then summed to give a squamous score; separate scores were similarly summed for fundic and pyloric glandular mucosa to give a glandular score. As findings were consistent for both methods of evaluation, real-time results were analysed because videos were missing or of inadequate quality for 11 examinations. Assessors were blinded to transport conditions and, for video analysis, to transportation vs confinement.

### 2.5. Haematology and Serum Biochemistry

Routine haematology and serum biochemistry parameters were determined as previously described [7]. Serum cortisol concentration was determined by radioimmunoassay (RIA) using the ImmunChemTM Cortisol 125 kit (MP Biomedicals, LLC, Orangeburg, NY, USA).

### 2.6. Statistical Analysis

All analyses were performed using SAS (SAS, version 9.4, 2018, Cary, NC, USA). For all statistical analyses, a *p*-value < 0.05 was considered significant.

#### 2.6.1. Effect of Confinement and Transport Conditions on Behavioural Parameters

As shown in Table 1, the frequency of each of identified behaviour in the ethogram during the 20 min observation window was recorded. None of the horses showed stereotypical behaviour; standing on three limbs and body stretching behaviours were observed only during confinement. Consequently, those behaviours were excluded in further analysis. Yawning and chewing/licking were not always visible during transportation and consequently were also excluded in further analysis. Total stress-related behaviour was calculated by summing the frequency of all behavioural events related to stress; total balance behaviour was calculated by summing the frequency of all behavioural events related to balance; total behavioural events were calculated by summing the frequency of all the single behavioural events recorded. All data were explored initially using summary statistics and normal distribution of all quantitative data was checked using the Anderson-Darling test. The effects of the three different feeding managements (feeding 12 h, 6 h or 1 h before loading) on behavioural data was tested using PROC mixed procedure and, because none of the models were significant, this factor was excluded in further analysis. Behavioural data were further analysed by three mixed linear models using PROC mixed procedure and each behavioural observation was analysed as a separate outcome variable. The first model evaluated treatment (transportation vs. confinement), hour (first, second to twelfth hour) and their interaction as fixed effects, with horse and replicate (day 1, day 2) as random factors. The second model compared space (stocks, single or wide bays) as a fixed factor, with horse, position (stocks, rear or forward facing) and replicate (day 1, day 2) as random factors. A third model was developed to identify behavioural differences due to the position (stocks vs. rear or forward facing) as a fixed factor, with horse, space (stocks, single and wide bays) and replicate (day 1, day 2) as random factors. A Tukey test was used as for post-hoc testing.

#### 2.6.2. Effects of Transport Conditions on Clinical, Laboratory and Gastroscopy Findings

The number of horses used in the present study was based on power analysis using previous results which suggested that n = 6 horses (the minimum sub-set in the experimental design) was sufficient to discriminate minimum differences in mean results of creatinine kinase, cortisol and gastric pH observed in similar studies [7]. Clinical, laboratory and gastroscopy data were analysed using PROC mixed procedure with treatment (transportation vs. confinement), time (T0, T1) and their interaction as fixed effects, with horse and replicate (day 1, day 2) as random factors, and each variable as a separate outcome. Clinical, laboratory and gastroscopy data were further analysed by two mixed linear models using PROC mixed procedure and each finding was analysed as a separate outcome variable. The first models were developed using space (single vs. wide bays), the time of sampling (T0, T1) and the interaction space*time as a fixed factors, with horse, position (rear and forward facing) and replicate (day 1, day 2) as random factors. The second model was developed using the position (rear or forward facing), time (T0, T1) and the interaction position × time as a fixed factor, with horse, space (single and wide bay) and replicate (day 1, day 2) as random factors. A Tukey test was used as for post-hoc testing.

#### 2.6.3. Associations among Behavioural, Clinical, Haematological, and Gastroscopy Parameters

Pearson correlations were calculated among behavioural, clinical, haematological and gastroscopy parameters. The behavioural parameters were expressed as the sum of the behaviour measured during the 12 h of confinement/journey. Clinical parameters, serum cortisol, haematological and serum biochemistry analytes were expressed as the difference between pre and post confinement/transportation results (T1–T0). Gastroscopy findings (i.e., squamous ulceration scores and glandular ulceration scores) were those recorded at the end of confinement/transportation (T1). Associations with a significant Pearson correlation (*p* < 0.05) were further investigated using univariate logistic regression analysis with clinically significant equine squamous gastric ulcer disease (ESGUS, defined as horses with a squamous ulcer score of ≥3 in any single location and/or a summed score of ≥5) as the binary outcome.

## 3. Results

### 3.1. Effect of Confinement and Transport Conditions on Behavioural Parameters

**Figure 3:**
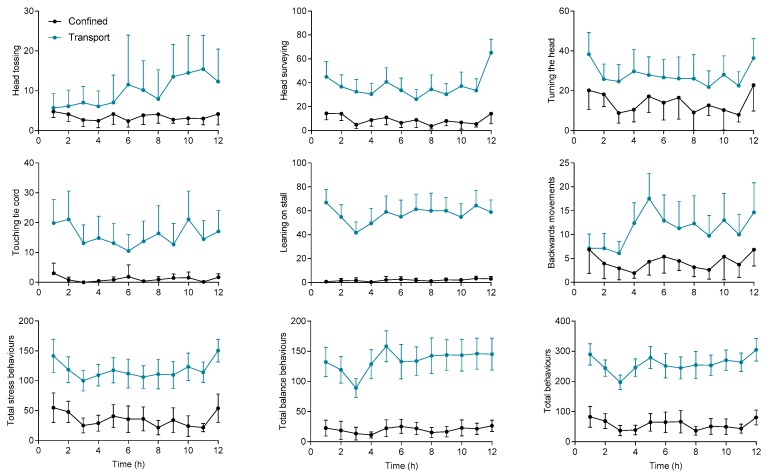
Frequency (n/20 min) of key behavioural parameters during the 12 h of transportation (blue) and confinement (black). Results are shown as mean and standard error.

Horses were very quiet during confinement. The average total behaviour, the summed frequency of the identified behaviours counted during the 20 minutes’ observation window over the 12 h of confinement, was about 700 behaviours/240 min (min 456, max 1457), fewer than 3 behaviours per minute. There were periods where horses were resting on three legs, showing a position and demeanour typical of sleep (i.e., neck below wither height, relaxation of the low lip, semi closure of both eyes, standing on three or four legs). Some horses lost their balance during sleeping, which was the only occasion this behaviour was observed during confinement. Sleeping periods were more often observed between 20:00 h and 22:00 h and between 12:00 h and 4:00 h. After a period of sleeping, horses typically exhibited body stretching. In contrast, horses during transport did not show any behaviours consistent with sleeping; they did not rest on three legs or stretch their body. At approximately 3300 events per 240 min (min 1795, max 5118), almost a behaviour every 4 s, the frequency of total behavioural events was greatly increased during transport. Figure 3 shows how the frequency of key behaviour recorded varied during the 12 h of confinement/transportation.

Transportation was associated with increased frequency of head-tossing (*p* = 0.002), head surveying (*p* < 0.001), turning the head (*p* < 0.001), touching the cord (*p* = 0.0001), leaning on the partitions (P=0.001), total balance behaviours (*p* = 0.008), total stress behaviours (*p* < 0.001) and total behavioural events (*p* = 0.008) in comparison with confined horses. In particular, leaning on the partitions, touching the tie cord, head surveying, total stress, total balance and total behaviour were consistently higher throughout the observation period. For confined horses, significant variation due to hours of confinement was evident only for turning the head (12th h vs. 4th–8th–10th–11th h), total stress (1st vs. 3rd–8th and 11th h) and total behaviour (1st and 12th h vs. 3rd–8th and 11th h); whereas in transported horses, a significant variation due to hours of journey was evident for all the studied behaviour except scratching, head tossing, lateral movements and licking the truck. A significant interaction between hours and treatment (confinement vs transportation) was observed for backward movements (*p* = 0.04) and head surveying (*p* = 0.026) (Table S1).

**Figure 4:**
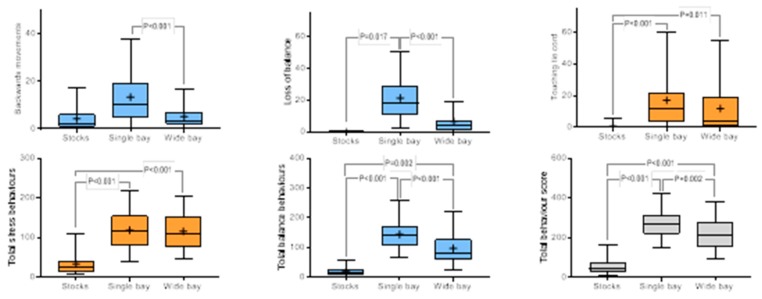
Effect of space (stocks, single or wide bays) on key behavioural characteristics. Results are shown as mean (₊) and median (horizontal line), quartile (box) and 95% confidence interval (whiskers), with significant differences between groups shown. Balance-related behaviours are blue, stress-related behaviours are orange, total behaviours are shown in grey.

The effects of space (single or wide bay) and position (rear or forward facing) were compared with observations from horses confined in reproductive stocks (Table 2; Table 3, respectively). Horses travelling in wide bays showed less leaning on the stall, less loss of balance, and fewer backward, and total balance movements in comparison with horses travelling in single bays. Horses travelling in single bays also showed more biting, head tossing, touching the tie cord and turning the head behaviour in comparison with horses travelling in wide bays and in confinement. Overall, horses travelling in single bays showed the highest frequency of total balance and total behaviour in comparison with horses both in wide bays and stocks. There was no difference in stress behaviours in single or wide bays, but the frequency of stress-related behaviour was least in stocks (Figure 4).

**Figure 5:**
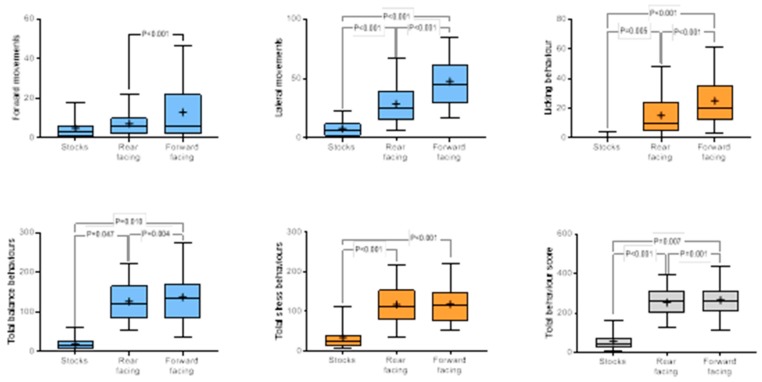
Effect of direction of travel (stocks, forward or rear facing) on key behavioural characteristics. Results are shown as mean (₊) and median (horizontal line), quartile (box) and 95% confidence interval (whiskers), with significant differences between groups indicated. Balance-related behaviours are blue, stress-related behaviours are orange, total behaviours are shown in grey.

Horses travelling in a forward-facing position showed more backward, forward and lateral movements, interaction, and licking, but less loss of balance, than horses travelling in a rear-facing position. The frequency of total balance behaviours and total behaviour was higher in horses travelling facing forward than horses in a rear-facing position or during confinement. No differences in total stress behaviours were evident between rear or forward-facing horses, but horses showed more stress-related behaviour during transportation than during confinement (Figure 5).

### 3.2. Effects of Transport Conditions on Clinical, Laboratory and Gastroscopy Findings

**Figure 6:**
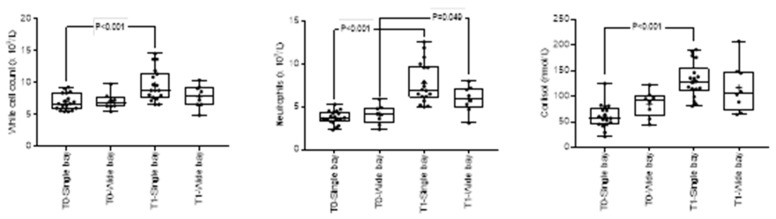
Effect of the interaction between space (single or wide bays) and time (T0, T1) on cortisol, white blood cell and neutrophil counts. Results are shown as mean (₊) and median (horizontal line), quartile (box) and range (whiskers), with all data points shown.

Transportation was associated with increases in heart rate and rectal temperature, reduced gastrointestinal borborygmi (decreased GI auscultation scores), changes in haematology and serum biochemistry and with increased gastric squamous ulcer scores (Table S2). There was a significant interaction between time (T0 vs. T1) and space (single and wide bays) only on cortisol (*p* = 0.017), white blood cell (*p* = 0.030) and neutrophil counts (*p* = 0.008) (Figure 6). Differences between single and wide bays were not significant at either time point. The direction of travel, time and their interaction had no significant effect on any of the variables tested.

### 3.3. Associations between Behavioural, Clinical and Gastroscopy Parameters

Significant correlations between observed behaviours and clinical parameters are presented in Table 4. Changes in heart rate after transportation or confinement (T1–T0) were positively correlated with behaviours relating to movement (lateral movement, leaning, loss of balance and total balance behaviours) or stress (licking, head surveying and total stress behaviour), and to the total behaviour score. Changes in rectal temperature after transportation were related to lateral movements, leaning, loss of balance, licking, interaction and head surveying. Weaker, but significant, associations were evident between stress behaviours and the change in GI activity score, such that horses exhibiting the greatest decrease in gastrointestinal borborygmi exhibited more frequent interactions, licking and total stress behaviours. Observed changes in CK were correlated positively with leaning and total balance, total stress and total behaviour scores; changes in AST were correlated positively with behaviours relating to movement (lateral movement, leaning, loss of balance and total balance behaviours) or stress (licking, head surveying, touching the cord, pawing, turning the head and total stress behaviour), and to the total behaviour score. Changes in cortisol were not associated with observed behaviours. The severity of squamous ulceration evident after transportation (T1) was positively correlated with leaning on partitions, loss of balance, licking, head surveying, total stress behaviours, total balance behaviours, and total behaviour; and a weak negative association was observed with head tossing. Contrariwise, the severity of glandular ulceration evident at T1 was correlated with none of the behavioural parameters.

In the univariate logistic model, loss of balance and changes in HR and RT were the only predictive variables which proved to be associated with a clinical ESGD (Table S3). For a unit increase in HR, RT, or loss of balance, the odds in favour of developing ESGD increased by a factor of 1, 5.4 and 1, respectively (Table 5).

## 4. Discussion

The current study compared behaviours in horses transported for 12 h with those observed in horses confined for a similar time period. The findings supported the main hypothesis that behaviours relating to stress and balance occurred more frequently in transported horses than in confined horses, and horses transported in a rear-facing position and in a wider bay size showed less balance-related behaviour. In particular, horses travelling in a single bay showed a higher frequency of behavioural events (both related to stress and balance) and, after the journey, demonstrated increases in cortisol, neutrophils and WBC that were not observed in horses travelling in wider bays. These observations suggest that during 12 hours’ transportation, rear-facing position and wider bays may reduce the impact of transport on horse health and welfare. The second hypothesis was partially supported, as balance-related behaviours, particularly loss of balance, were associated with the severity of gastric ulceration and the increase in muscle enzymes after transportation, while stress behaviours (licking and total stress behaviours) correlated with decreased gastrointestinal sounds but not with cortisol. Increases in heart rate and rectal temperature observed after transportation were also positively associated with balance and stress behaviours, and with the development of clinical ESGD in the univariate regression model. These findings suggest that behaviour in transit and physiological measurements after transportation might identify horses at risk for development of transport-related disease and, for the first time, this study documented the effects of space during a long journey on behaviour, health and physiological parameters. Adequate space allowance and rear-facing positioning would appear to facilitate better balance, but effects of bay size and direction of travel on stress behaviours and long-term outcomes were less clear and require further study.

Transportation is considered stressful because horses are confined in a small space [33,34]. However, in our study, horses showed a different behavioural repertoire during confinement and transportation. Transportation was associated with increased head movements (head tossing, turning and surveying), as well as with increased touching of the tie cord, relative to behaviours observed during confinement. The observed head movements were interpreted as indicative of increased arousal (i.e., anxiety/vigilance/alert response). Touching the tie cord during transportation has been previously correlated with cortisol [5], and it may be interpreted as a redirected behaviour [27]; the horse would like to do something else but instead starts touching the tie cord, which often is the only behaviour it is free to do. During transport, the horses repeatedly licked the surface in front of them. Licking in this manner may be considered as an oral stereotypy, because it was abnormal in its frequency and with no obvious purpose (there was no evidence that horses were seeking feed material or other substances such as salt). Both licking and touching the tie cord were behaviours observed infrequently during confinement and have been well characterised as indicators of stress [27]. Unfortunately, chewing/licking, another behaviour associated with stress [35], was excluded from the current study because available camera angles inconsistently precluded continuous observation of these behaviours during transportation, and we cannot confirm previous results which licking was positively correlated with the time horses spent with the head in a upper position [5]. The high frequency of behaviours related to stress and the high frequency of total behaviours, demonstrate that transportation was much more stressful than confinement for our horses, confirming that transport stress is multifactorial [36]. In our study, confined horses appeared to be able to keep their circadian pattern of sleeping, at least the standing sleep (low-wave sleep), intermittently. This was not possible for the horses during transportation. Overnight travel is recommended during summer to minimise the effects of high ambient temperatures, but there are currently no studies on the effects of sleep deprivation due to transport in horses. As sleep deprivation in other species has been associated with altered immune function [37] and with impaired athletic performance [38,39], the physiological implications of travelling overnight warrant further evaluation.

As expected, transportation was associated with increased balance behaviours (learning on rails, forward, backwards and lateral movements) in comparison with confinement. It is well recognised that maintaining balance in a truck is difficult, causing an increase in muscle enzymes [25,40], and that factors like vehicle design, type of suspension, driver ability and type of road all have an effect on the horse’s ability to balance with more or less effort [11,41,42]. The truck used in the current study was equipped with good suspension and a driver of many years of experience in live animal transportation, but the chosen route was a mix of minor and major roads, with straight and winding sections to mimic commercial transport routes. The variations of balance-related behaviour throughout the journey, in particular their decreased frequency during the third hour of transport, may therefore be related more on the type of road and route (a straight tract on a major road) than to other animal-related factors. Instead the high frequency of balance-related behaviour during the first and the last hour may be related to both route and arousal conditions. Horses can sense when they are close to their home range or stable and can get more restless during the last part of the trip. The horses tended to move more when aroused, a trend which was also evident during confinement, where the horses showed more turning the head, total stress and total behaviour during the first and the last hour and exhibited postures associated with increased arousal such as head elevation and pricking of the ears. The observed changes in haematological, blood biochemistry, cortisol concentrations and squamous ulcer scores observed in the current study were expected and are consistent with previous studies [7,43,44,45], and may be explained by the higher arousal and difficulty of maintaining balance observed during transit in comparison with confinement.

The novelty of this study was the effects of space on the behaviour, health and welfare of the transported horses. Horses travelling in wider bays showed less balance-related movements, leaning less on the partitions and losing their balance less often, and less behaviour related to vigilance, possibly because their arousal was lower. Studies have demonstrated that horses experiencing loss of balance, scrambling, abrupt braking and cornering were more agitated and anxious during the journey, possibly due to fear of falling inside the trailer [46]. The recommended loading density for horses loaded in groups is y = (54.837) × W^0.325^, where y = density in kg/m^2^ and W = average animal weight in kilograms, about 1.2 m^2^ for a 500 kg horse [16]. However, the latter study considered slaughter horses and injuries as the only welfare outcome evaluated. Minimal space allowance for an adult horse during a long journey is 1.75 m^2^ in Europe [19], or 1.2 m^2^ in Australia [20]. However, in many other countries minimal space allowance is not reported or a general and vague recommendation in line with the OIE regulation is provided (i.e., horses should have sufficient space to adopt a balanced position as appropriate to the climate and species transported) [47]. The most recent European guidelines on transport of slaughter horses, derived from a Delphi survey method, suggest that horses should be transported with 10 and 20 cm of total space between animal and partitions [48,49]. This is the first study to report animal-based evidence suggesting that horses travelling in a wide bay of 1.9 m^2^ are better able to balance, minimising the implications of transport on behaviour, health and welfare. Thus, findings may be useful for updating standards related to the minimum space allowances required for horses during long journeys.

Facing away from the direction of travel has been recommended in the literature [12,50,51]. However, the majority of horse trailers and trucks are built to transport horses facing forward, and results from available studies are conflicting. Rear facing has been previously reported to be associated with fewer impacts against the slides and ends of the trailers, less frequent loss of balance, fewer total behaviour and less balance movement [12,15,50,52]. Smith et al. [51] found that horses travelling untethered preferred to travel facing backward, and Kusunose et al. [53] confirmed this preference, demonstrating that yearlings learn quickly that facing backwards is advantageous during transport. However, these differences were not observed in other studies, and it was reported that the preference in the direction of travel is individual and may be related to the past experience [54]. In the current study, rearward facing horses demonstrated fewer backwards, forwards and lateral movements and less total balance behaviour, as hypothesised. However, unexpectedly, rear-facing horses also demonstrated increased loss of balance in the current study. The latter results may be due to other factors, such as truck configuration, and therefore require clarification. Horses transported facing forwards demonstrated increased licking behaviour, increased interactions and total behaviour compared to rear facing and confined horses. This may be due to their difficulty maintaining balance which might increase anxiety and induce increased redirected behaviours and looking for a social calming effect. The fact that horses travelling facing the direction of travel are aroused is in line with the literature, where it was demonstrated that heart rate, HRV and salivary cortisol were higher in horses facing forward after a journey of short and middle duration [44]. The current study demonstrated no significant effects attributable to direction of travel on clinical parameters (heart rate, respiratory rate, rectal temperature, GI auscultation scores), haematology or serum biochemistry, plasma cortisol or gastric squamous or glandular ulcer scores. This may therefore suggest that direction of travel is important for the ability of horses to keep their balance and may minimise anxiety, but it is does not substantially affect health.

Correlations were observed between several behavioural parameters and physiological parameters measured in the current study. Changes in heart rate and in rectal temperature (i.e., the difference between values obtained prior to departure and on return from travel) were correlated with total stress behaviours, total balance behaviours and the total behaviour score. This may be due to the strong connection between behaviour, sympathetic system and thermoregulation [55]. Similarly, the correlations between frequency of stress behaviours, interaction and licking with decreased GI motility may suggest that these behaviours are associated with both increased sympathetic tone and, therefore, with decreased GI motility. It has been reported that transportation causes decreased gut sounds [5,24] and increased risk of colic, in particular colon impaction [56]; however, more studies on this relationship are needed. Importantly the severity of ESGD was associated with several balance behaviours, in particular loss of balance. Gastroscopic observations reported previously for these horses suggested that ulceration was likely due to contact between the squamous mucosa and alkaline gastric fluid, and it is likely that the increased loss of balance observed in the current study facilitated such contact. The severity of ESGD after transportation was also correlated with stress-related behaviour, head surveying, tossing and licking. Horses with gastric squamous ulceration tended to be more reactive to a novel test, spend more time away from the novel object, pawed more and tended to show oral stereotypy [57]. However, in the same paper, authors failed to demonstrate a difference in pain-related behaviour, heart rate, cortisol and other clinical parameters between horses with and without stomach ulcers. Similarly, we were not able to find any associations between glandular ulcers and behaviour, haematology or situations of travel. This might reflect the mild and clinically insignificant glandular lesions observed in the current study, or could be related to the difficult and still unclear aetiology of glandular ulcers [58]. We also failed to find associations between behaviour and haematology or biochemistry changes, or with transport conditions tested (width of bay or direction of travel). This was surprising, as it was hypothesised that increased stress behaviours would be associated with increased cortisol concentrations and increased movement with increased muscles enzymes. Our findings suggest that behavioural changes and non-invasive measures of autonomic balance such as heart rate or heart rate variability and GI motility might be more sensitive indicators of horses’ response to transportation than haematology, biochemistry or endocrine parameters. Consequently, our study suggests that monitoring behaviour and physiological responses in transit and after transportation are likely to identify horses that are at risk for transport-associated disease.

Our results need to be interpreted with caution, because the study was limited by several factors. First of all, the number of horses travelling in wide bays was lower than those travelling in single bays, due to restrictions in the configuration of the truck. The truck configuration also prevented a balanced study design relating to the number of horses travelling backwards and forwards. The preferences and travel history of each horse were unknown, and we were unable to control for this, for example by use of a repeated measures or cross-over study. The truck also had different compartments, and the first compartment, located over the trailer’s connection to the prime mover, and where two horses were located in rear facing wide bays, was considered the least stable due to its height and the rotational forces during turning. Consequently, the configuration of the truck may have confounded our results, and the frequency of total balance behaviour reported by horses in wide bays and rear facing may be overestimated. Conversely, horses used in the confinement study and consequently fasted 12 h before the journey were all located in the two rear-most compartments. Random allocation into different compartments and bay sizes would have been a more desirable strategy, but this would have prevented other study outcomes. An equal number of horses used in confinement travelled forwards as backwards, and behaviours in this group were not significantly different from those observed in other horses, suggesting that feeding management prior to departure did not influence observed behaviours. Only a confinement/transportation of 12 h were tested, so our results may not be repeated in journeys of shorter duration. Finally, some behaviours, such as the frequency of yawning, chewing and lip smacking were not always visible during transportation and had to be excluded from the analysis. Notwithstanding those limitations, this study has reported behavioural changes associated with space allowance and direction of travel, and is the first study reporting the effects of a wider bay on behaviour and health of horses in comparison with single bay or confinement alone. As such, it has increased our knowledge of transport stress and how to mitigate it.

## 5. Conclusions

This study documented that travelling in a wide bay was advantageous for the horses, since they could balance better and demonstrated fewer anxiety-related behaviours than horses travelling in single bays. A positive effect on balance was also seen in the horses travelling facing away from the direction of transport. The number of movements, leaning on the partitions, and loss of balance were related to the risk of development gastric squamous ulceration during transportation. As behaviour was more sensitive than haematology, biochemistry or plasma cortisol for assessing the emotional status of the animals in transit, video-cameras for observing the behaviour of horses during transportation are strongly recommended. Further studies on horse preference of direction of travel and the effect of space and direction of travel on respiratory and gastrointestinal diseases related to transportation are recommended to confirm these results and to identify mechanisms to minimise adverse impacts of transport stress on horse behaviour, health and welfare.

## Figures and Tables

**Table 1:** Behaviour sampling ethogram used to measure the frequency of selected behavioural events during confinement or transportation. Total stress-related behaviour was calculated summing the frequency of all behavioural events related to stress. Total balance behaviour was calculated summing the frequency of all behavioural events related to balance. Total behavioural events were calculated summing the frequency of all the single behavioural events.

Behaviour	Description
Behavioural events related to stress (Expressed as frequency) (n/20 min)	
Biting neighbour	The horse bites the neighbour
Explorative behaviour/sniffing	The horse sniffs around, it sniffs some area of the truck/box
Head Surveying	Head scanning through forty-five degrees or more, ears pricked up pointing forwards and stationary for 3 s or more (adapted from [26])
Head tossing/shaking	The horse shakes its head suddenly, violently and frequently [5]
Chewing/licking	Opening of mouth with extension and retraction of tongue, lip smacking without tongue extension, lateral jaw movements involving partial opening of the lips [27]
Licking the truck/wall	The horse licks part of the truck/box (wall, stall rails)
Pawing	One front leg is lifted from the ground slightly, then extended quickly in a forward direction, followed by a movement backward, dragging the toe against the floor in a digging motion [28]
Scratching	Rubbing any part of the body against part of the stock/truck (Adapted from [26]
Stereotypy	The persistent repetition of a behaviour for no obvious purpose [27]
Touching tie cord	The horse touches the rubber cord with which he is tied [5] in the truck or in the stocks
Turning the head	The horse turns his head and neck to the right or to the left appearing to look at his flank
Total stress-related behaviours	Sum of the behavioural events related to stress
Behavioural events related to balance (Expressed as frequency) (n/20 min)	
Backward movements	The horse steps backward
Forward movement	The horse steps forward
Lateral movements	The horse steps sideways
Leaning on stall rails	The horse gently leans laterally against one of the two stall rails of the stock or of the bay
Loss of balance/dashing on the partitions	The horse losses his balance and crashes/bumps on one stall rails
Total balance-related behaviours	Sum of the behavioural events related to balance
Other behavioural events (Expressed as frequency) (n/20 min)	
Interaction with neighbours	The horse interacts with one of his neighbours through the stall rails, they sniff each other
Stand on three limbs	The horse is standing on 3 or 4 limbs without moving in any direction [29]
Body stretching	Rigid extension of the limbs and arching of the neck and back [26]
Yawning	An involuntary sequence consisting of mouth opening, deep inspiration, brief apnoea, and slow expiration [30]
Total behavioural events	Sum of all behavioural events

**Table 2:** Effect of space (stocks, single or wide bays) on the studied behaviours. Data are expressed as the least square mean and standard error (SE) of the number of observed behavioural events, with *p*-value determined by linear mixed model and Tukey post-hoc testing. Means with different superscript differ significantly (A, B, C, *p* < 0.01; a, b, *p* < 0.05).

Behaviour	Single Bay	Wide Bay	Stock	*p* Value
Backward movement	14.9 ± 4.9 ^A^	5.2 ± 5.1 ^B^	4.3 ± 6.8 ^A,B^	0.002
Biting	4.1 ± 0.7 ^A^	0 ± 1.1 ^B^	0 ± 0.8 ^B^	<0.001
Explorative behaviour	1.2 ± 0.7	2.6 ± 0.8	2.1 ± 0.9	ns
Forward movements	13.7 ± 5.6 ^A^	4.0 ± 5.8 ^B^	4.9 ± 7.7 ^A,B^	0.007
Scratching	2.4 ± 0.5	1.8 ± 0.9	1.9 ± 0.6	ns
Head surveying	35.5 ± 3.5 ^A^	43.1 ± 5.4 ^A^	6.9 ± 3.7 ^B^	<0.001
Head tossing	10.1 ± 1.4 ^A^	7.7 ± 2.2	4.3 ± 1.5 ^B^	0.006
Interaction	9.8 ± 3.2	8.5 ± 3.6	4.47 ± 4.2	ns
Lateral movements	38.3 ± 10.9	38.3 ± 11.2	8.3 ± 15.3	ns
Leaning on the stall rails	62.9 ± 3.6 ^A^	43.0 ± 5.5 ^B^	2.5 ± 3.7 ^C^	<0.001
Licking	21.9 ± 6.4	16.8 ± 6.7	2.5 ± 8.8	ns
Loss of balance	19.9 ± 5.2 ^A,a^	6.6 ± 5.4 ^B^	0 ± 7.2 ^b^	<0.001
Pawing	0.5 ± 0.5	1.1 ± 0.7	1.1 ± 0.5	ns
Touching the tie cord	17.1 ± 2.4 ^A^	10.9 ± 3.6 ^a^	0 ± 2.5 ^B,b^	<0.001
Turning the head	27.6 ± 3.9 ^A^	28.1 ± 5.9	17.7 ± 4.0 ^B^	<0.001
Total behaviour	278.5 ± 21.5 ^A^	217.5 ± 24.7 ^B^	59.1 ±28.7 ^C^	<0.001
Total stress	119.0 ± 7.2 ^A^	112.2 ± 10.2 ^A^	35.6 ± 7.3 ^B^	<0.001
Total balance	148.7 ± 13.9 ^A^	96.2 ± 15.5 ^B^	18.2 ± 18.7 ^C^	<0.001

**Table 3:** The effect of positioning (rear- and forward-facing and stock) on observed behaviours. Data are expressed as the least square mean and standard error (SE), with *p*-value determined by linear mixed model and Tukey post-hoc testing. Means with different superscript differ significantly (A, B, C, *p* < 0.01; a, b, *p* < 0.05).

Behaviour	Rear Facing	Forward Facing	Stocks	*p*-Value
Backward movement	5.3 ± 4.8 ^A^	15.1 ± 4.9 ^B^	4.3 ± 6.7	<0.001
Biting	2.1 ± 2.2	1.9 ± 2.1	0.1 ± 3.0	ns
Explorative behaviour	2.6 ± 0.8	1.3 ± 0.8	2.1 ± 1.0	ns
Forward movements	3.6 ± 4.7 ^A^	14.5 ± 4.8 ^B^	4.8 ± 6.6	<0.001
Scratching	2.4 ± 0.6	2.0 ± 0.7	1.8 ± 0.6	ns
Head surveying	39.6 ± 4.1 ^A^	36.1 ± 4.3 ^A^	7.8 ± 4.7 ^B^	<0.001
Head tossing	9.5 ± 1.4 ^A^	9.2 ± 1.7 ^A^	4.1 ± 1.5 ^B^	<0.001
Interaction	6.4 ± 1.3 ^A,a^	12.2 ± 1.4 ^B^	4.1 ± 1.3 ^A,b^	0.000
Lateral movements	26.8 ± 2.1 ^A^	48.9 ± 2.4 ^B^	8.0 ± 2.1 ^C^	<0.001
Leaning on the stall rails	56.2 ± 9.2 ^A^	51.1 ±9.3 ^A^	1.2 ± 12.4 ^B^	0.001
Licking	13.5 ±2.7 ^A^	25.9 ± 2.8 ^B^	2.1 ±3.4 ^C^	<0.001
Loss of balance	18.6 ± 6.5 ^A^	8.3 ± 6.5 ^B^	0 ± 9.2	<0.001
Pawing	0.9 ± 0.5	0.4 ± 0.6	1.2 ± 0.5	ns
Touching the tie cord	15.4 ± 2.8 ^A^	14.3 ± 3.1 ^A^	0 ± 3.3 ^B^	<0.001
Turning the head	28.1 ± 3.4 ^A^	27.9 ± 3.7 ^A^	18.0 ± 3.4 ^B^	<0.001
Total behaviour	230.7 ± 30.4 ^A^	269.2 ± 30.7 ^B^	57.8 ±41.7 ^C^	<0.001
Total stress	115.2 ± 6.5 ^A^	121.0 ± 7.4 ^A^	34.7 ± 6.5 ^B^	<0.001
Total balance	109.4 ± 26.9 ^A^	135.2 ± 27.1 ^B,a^	18.0 ± 37.5 ^b^	0.002

**Table 4:** Pearson correlations between the sum of the behavioural events recorded during transport and confinement, and observed changes (∆, T1–T0) in heart rate (HR), rectal temperature (RT) GI activity scores, CK and AST concentrations, and summed squamous ulcer scores at T1 (after confinement/journey). Results are expressed as r- and *p*-values.

Behavioural Parameters	∆ HR	∆ RT	Decrease in GI Activity Score	∆ CK	∆ AST	T1 SQ Score
Lateral movement	*p* = 0.001 r = 0.524	*p* = 0.015 r = 0.399			*p* = 0.041 r = 0.357	*p* = 0.056 r = 0.32
Leaning	*p* = 0.002 r = 0.587	*p* < 0.001 r = 0.692		*p* = 0.040 r = 0.357	*p* = 0.000 r = 0.611	*p* < 0.001 r = 0.544
Loss of balance	*p* < 0.001 r = 0.590	*p* < 0.001 r = 0.762			*p* < 0.001 r = 0.630	*p* < 0.001 r = 0.704
Licking	*p* = 0.004 r = 0.468	*p* = 0.001 r = 0.525	*p* = 0.009 r = 0.426		*p* = 0.004 r = 0.479	*p* = 0.016 r = 0.398
Interaction		*p* = 0.044 r = 0.337	*p* = 0.013 r = 0.408			
Head surveying	*p* = 0.002 r = 0.486	*p* < 0.001 r = 0.572			*p* = 0.005 r = 0.469	*p* = 0.046 r = 0.335
Head tossing						*p* = 0.026 r = -0.372
Total stress behaviour	*p* < 0.001 r = 0.657		*p* = 0.009 r = 0.427	*p* = 0.040 r = 0.353	*p* < 0.001 r = 0.718	*p* = 0.010 r = 0.424
Total balance behaviour	*p* = 0.001 r = 0.595			*p* = 0.043 r = 0.349	*p* = 0.000 r = 0.571	*p* = 0.001 r = 0.509
Total behaviour	*p* < 0.001 r = 0.675			*p* = 0.027 r = 0.383	*p* <0.001 r = 0.705	*p* = 0.001 r = 0.515

**Table 5:** Results of univariate regression analysis between clinical equine squamous glandular disease (ESGD, defined as horses with a squamous ulcer score ≥3 in any single location and/or a summed score ≥5 after transportation) as the binary outcome, loss of balance, changes in heart rate (HR) and changes in rectal temperature (RT) before and after transportation or confinement. Data were collected from confined (n = 12) and transported horses (n = 26).

Variable	Estimate	SE	OR	95% CI	*p*
HR	0.071	0.03	1.0	1.01–1.14	0.021
RT	1.688	0.74	5.4	1.26–23.10	0.023
Loss of balance	0.006	0.00	1.0	1.0–1.01	0.031

Legend: SE: standard error; OR: odds ratio; CI: confidence interval.

## Supplementary Materials

The following are available online at https://www.mdpi.com/2076-2615/10/1/160/s1, Table S1: Effect of treatment (transportation vs. stock), hour (first, second to twelfth hour) and their interaction as fixed effects on the studied behaviour. Data are expressed as the least square mean and standard error (SE), with *p* determined by linear mixed model and Tukey post-hoc testing. Means on the same line with different superscript differ significantly (A, B, C, D, E, F, *p* < 0.01); means on the same column with different superscript differ significantly (e, f, *p* < 0.05). Table S2: Effect of treatment (transportation vs stock), time (before (T0) vs. after (T1) transportation or stock) and their interaction as fixed effects on the clinical parameters. Data are expressed as the least square mean and standard error (SE), with *p* determined by linear mixed model and Tukey post-hoc testing. Means on the same line with different superscript differ significantly (A, B, *p* < 0.01; a, b, *p* < 0.05); means on the same column with different superscript differ significantly (E, F, *p* < 0.01; e, f, *p* < 0.05). Table S3: Wald test *p*-values generated from univariate regression analysis.
