# Supplementary material for: Effects of Transport Conditions on Behavioural and Physiological Responses of Horses
Source: Animals (Basel). 2020 Jan 17;10(1):160. doi: 10.3390/ani10010160 (PMC7022509; doi:10.3390/ani10010160)
Supplement: Supplementary file 1 [file animals-10-00160-s001.pdf]

Table S1: Effect of treatment (T)(transportation vs stock), hour (H)(first, second to twelfth hour) and their interaction (I) as fixed effects on the studied behaviour. Data are expressed as the least square mean and standard error (SE), with P value determined by linear mixed model and Tukey post-hoc testing. Means on the same line with different superscript differ significantly (A, B, C, D, E, F P<0.01); means on the same column with different superscript differ significantly (e,f P<0.05)

Behavior	Treatment	H1	H2	H 3	H4	H5	H6	H7	H8	H9	H10	H11	H12	SE	P (T)	P (H)	P (I)
Backwards movement	Stock	6.9	3.8	2.8	1.8	4.2	5.2	4.3	3.0	2.5	5.2	3.5	6.7	9.6	0.599	0.004	0.046
	Journey	6.7^{AD}	6.4^{ACD}	5.4^{D}	11.3	16.2^{E}	11.8^{EF}	10.2^{F}	11.2^{F}	8.6^{ABF}	11.9^{B}	9.3^{AC}	14.0^{BEF}	6.8			
Explorative behavior	Stock	3.7^{A}	3.2	2.1	0.7^{B}	2.6	2.6	3.6^{A}	0.8^{B}	1.3	1.5	1.5	2.3	1.5	0.923	0.001	0.469
	Journey	5.5^{A}	2.7^{AB}	2.1	2.0	3.6^{AC}	0.9^{BD}	1.2^{B}	0.6^{D}	0.7^{D}	1.5^{BC}	0.7^{C}	2.7^{ABC}	1.1			
Foreword movement	Stock	7.4	4.3	2.6	2.6	4.8	6.3	4.7	3.5	2.6	6.7	4.5	6.6	10.1	0.718	0.035	0.388
	Journey	6.2^{AC}	6.1^{C}	5.5^{C}	9.6	13.9^{B}	10.7	9.1	9.8	7.6	10.5	8.1^{ADE}	12.4^{ABE}	7.2			
Scratching	Stock	2.1	2.2	1.0	2.0	1.4	2.4	0.7	1.6	6.3	0.7	0.3	1.7	1.6	0.504	0.457	0.676
	Journey	4.3	4.1	1.7	1.7	1.2	1.9	1.7	2.8	2.2	2.9	1.6	1.3				
Head surveying	Stock	13.7^{e}	13.9^{e}	4.6^{e}	8.5^{e}	10.7^{e}	6.2^{e}	8.7^{e}	3.5^{e}	7.7^{e}	6.5^{e}	5.2^{e}	13.9^{e}	5.8	<0.001	<0.001	0.025
	Journey	45.1^{Af}	36.9^{ABDf}	32.7^{BDf}	31.4^{BDf}	41.6^{ABDf}	34.6^{ABDf}	27.1^{Df}	35.4^{ABDf}	31.2^{BDf}	38.1^{ABDf}	33.6^{ABDf}	65.4^{Cf}	4.5			
Head tossing	Stock	5.1	4.5	3.3	3.0	4.7	2.9	4.4	4.7	3.4^{e}	3.7^{e}	3.6^{e}	4.7	3.9	0.001	0.898	0.771
	Journey	5.7	6.1	7.0	5.8	6.8	11.3	9.9	7.7	13.3^{f}	14.3^{f}	15.4^{f}	12.3	2.8			
Interaction	Stock	6.5	4.8	2.8^{A}	1.7	4.7	5.2	9.5^{B}	0.6^{A}	1.6^{A}	2.8^{A}	2.8^{A}	5.5	4.4	0.263	0.025	0.414
	Journey	9.2	8.1	9.6	9.4	8.7	12.3^{A}	9.5	8.5	7.3	9.7^{B}	7.8	11.7	3.2			
Lateral movement	Stock	8.9	8.7	8.1	5.4	9.6	9.7	9.3	6.5	8.0	7.0	9.0	9.2	15.7	0.107	0.166	0.375
	Journey	37.3	38.1	28.1	40.3	39.1	30.9	33.4	37.1	43.0	40.3	44.7	45.1	11.2			
Leaning	Stock	1.3^{e}	1.2^{e}	1.3^{f}	0.1^{e}	1.9^{e}	2.3^{e}	1.5^{e}	1.0^{e}	1.9^{e}	1.7^{e}	3.0^{e}	2.7^{e}	14.1	0.001	0.187	0.342
	Journey	63.4^{Af}	51.3^{Af}	38.2^{Bf}	45.9$^{A f}$	55.4^{Af}	51.3$^{A f}$	57.6$^{A f}$	56.3$^{A f}$	56.4$^{A f}$	51.2^{Af}	60.9^{Af}	55.4^{Af}	10.1			
Licking	Stock	2.5	1.8	1.7	1.9	2.1	3.0	2.0	1.6	1.8	1.7	1.4	2.3	9.5	0.102	0.781	0.803
	Journey	17.7	20.2	16.5	22.1	22.9	21.4	25.3	21.7	21.8	15.7	18.4	15.0	6.7			
Loss of balance	Stock	0	0	0	0	0	0	0	0	0	0	0	0	11.8	0.296	0.076	0.088
	Journey	20.5^{AD}	10.5^{BC}	5.0^{C}	13.1^{AB}	20.6^{D}	15.3^{BD}	11.2^{BC}	13.1^{BC}	13.6^{BD}	16.1^{ABD}	13.4^{BD}	10.9^{BD}	8.3			
Pawing	Stock	3.4	1.7	0.6	0.7	0.8	3.0	1.1	0.8	0.4	0.6	1.4	0.8	1.2	0.201	0.024	0.945
	Journey	4.6^{A}	0.9^{B}	0.4^{B}	0.6^{B}	0.6^{B}	0^{B}	0.3^{B}	0.1^{B}	0^{B}	0^{B}	0.3^{B}	0.4^{B}	0.9			
Touching tie rope	Stock	0.2^{e}	0^{e}	0^{e}	0^{e}	0^{e}	0^{e}	0^{e}	0^{e}	0^{e}	0^{e}	0^{e}	0^{e}	4.8	0.001	0.505	0.692
	Journey	19.9^{ABf}	20.5^{Bf}	12.5^{f}	13.7^{f}	12.0^{f}	9.4^{Cf}	12.6^{f}	15.2^{f}	11.5^{f}	13.9^{ABf}	16.4^{f}	20.5^{f}	3.6			
Turning the head	Stock	24.3^{e}	22.1	12.7^{Ae}	14.4^{Ae}	21.1	17.9	20.4	13.^{0Ae}	16.1	14.2^{Ae}	11.8^{Ae}	26.7^{B}	5.2	<0.001	0.001	0.838
	Journey	38.3^{Af}	25.8^{B}	24.7$^{B f}$	29.8^{f}	27.9^{B}	26.7	26.2	26.1^{f}	21.9	28.1^{f}	22.5^{f}	36.4^{A}	4.2			
Total behaviour	Stock	84.1^{Ae}	68.9^{e}	37.3^{Be}	39.2^{e}	64.9^{e}	65.0^{e}	66.7^{e}	36.8^{Be}	50.7^{e}	49.8^{e}	43.9^{e}	80.7^{Ae}	50.7	0.001	<0.001	0.333
	Journey	283.7^{Af}	238.0^{B}	190.9^{B}	236.6^{B}	269.4	241.9^{B}	235.1^{B}	244.8^{B}	244.0^{B}	260.8^{B}	257.2^{B}	298.7^{A}	36.1			
Total stress	Stock	52.2^{Ae}	45.7^{e}	22.2^{Be}	27.2^{e}	39.5^{e}	35.7^{e}	36.8^{e}	21.7^{Be}	33.8^{e}	25.8^{e}	20.6^{Be}	49.5^{e}	11.9	<0.001	0.001	0.792
	Journey	141.6^{ACf}	118.4^{f}	100.1^{Bf}	108.6^{Bf}	116.8^{f}	111.0^{Bf}	105.4^{Bf}	110.2^{Bf}	108.9^{Bf}	122.4^{ABf}	114.1^{ABf}	150.4^{Cf}	9.1			
Total balance	Stock	20.7^{e}	14.4^{e}	8.2^{e}	6.2^{e}	16.8^{e}	20.1^{e}	16.3^{e}	10.4^{e}	11.3^{e}	17.2^{e}	16.5^{e}	21.7^{e}	34.9	0.008	0.023	0.345
	Journey	126.4^{ACf}	109.6^{Cf}	79.2^{Bf}	117.7^{ACf}	142.9^{Af}	117.6^{ACf}	119.2^{ACf}	125.1^{ACf}	126.8^{ACf}	127.6^{ACf}	133.4^{ACf}	134.7^{Af}	24.9			

Table S2: Effect of treatment (transportation vs stock), time (before (T0) vs after (T1) transportation or stock) and their interaction (I) as fixed effects on the clinical parameters. Data are expressed as the least square mean and standard error (SE), with P value determined by linear mixed model and Tukey post-hoc testing. Means on the same line with different superscript differ significantly (A, B, P<0.01; a, b P<0.05); means on the same column with different superscript differ significantly (E, F P<0.01; e, f P<0.05)

**Paramter**	**Treatment**	**T0**	**T1**	**SE**	P (T)	P (H)	P (I)
**Body weight**	Stock	$527.8^{A,e}$	513^{B}	12.5	0.101	<0.001	0.024
	Journey	518.8^{f}	514.4	12.4			
**Hear rate**	Stock	38.6	36.3^{E}	2.3	0.242	0.002	<0.001
	Journey	32.7^{A}	$46.9^{B,F}$	1.7			
**Respiratory rate**	Stock	9.8	10.9	1.4	0.012	0.289	0.839
	Journey	12.7	14.3	1.0			
**Rectal Temperature**	Stock	37.3^{e}	37.4^{e}	0.1	0.957	<0.001	<0.001
	Journey	$37.0^{A,f}$	$37.8^{B,f}$	0.1			
**Capillary refilling time**	Stock	$1.0^{A,E}$	1.6^{B}	0.1	0.030	0.001	<0.001
	Journey	1.5^{F}	1.4	0.1			
**Gut sounds**	Stock	7.9^{A}	$6.5^{B,E}$	0.3	0.005	<0.001	0.002
	Journey	8.0^{A}	$4.8^{B,F}$	0.2			
**Sum Squamous ulcerations**	Stock	0.6	1.4^{E}	0.6	0.003	<0.001	0.009
	Journey	1.2^{A}	$4.5^{B,F}$	0.4			
**Sum Glandular ulcerations**	Stock	1.0	1.3	0.2	0.862	0.416	0.416
	Journey	1.2	1.2	0.2			
**RBC**	Stock	8.2^{a}	7.5^{b}	0.3	0.821	0.308	0.001
	Journey	7.6	8.0	0.2			
**HCT**	Stock	38.0^{a}	$32.^{0be}$	0.0	0.504	0.140	0.002
	Journey	34.8	37.0^{f}	0.0			
**WBC**	Stock	8.2^{a}	$6.7^{b,E}$	0.4	0.089	0.307	<0.001
	Journey	7.0^{A}	$9.0^{B,F}$	0.3			
**Neutrophils (N)**	Stock	4.6	4.6^{E}	0.4	0.011	<0.001	<0.001
	Journey	3.8^{A}	$7.2^{B,F}$	0.3			
**Lymphocytes (L)**	Stock	3.1^{A}	1.8^{B}	0.2	0.020	<0.001	0.631
	Journey	2.7^{A}	1.5^{B}	0.1			
**N/L**	Stock	1.5	2.8^{E}	0.5	0.002	<0.001	0.002
	Journey	1.5^{A}	$5.6^{B,F}$	0.4			
**Fibrinogen**	Stock	2.0	2.1	0.1	0.210	0.025	0.378
	Journey	2.0^{a}	2.3^{b}	0.1			
**Total proteins**	Stock	71.1^{E}	74.1	1.2	0.061	<0.001	0.003
	Journey	$67.1^{A,F}$	74.8^{B}	0.9			

**Albumin**	Stock	33.6^{E}	34.7	0.4	0.018	<0.001	<0.001
	Journey	$31.5^{A,F}$	35.3^{B}	0.3			
**CK**	Stock	307.8	304.2^{e}	60.9	0.019	0.124	0.108
	Journey	353.3^{a}	$512.2^{b,f}$	44.1			
**AST**	Stock	277.9	282.7	8.1	0.887	0.001	0.003
	Journey	261.1^{A}	301.4^{B}	6.2			
**Na**	Stock	134.8	135.2	0.4	0.834	0.207	0.544
	Journey	134.6	135.3	0.4			
**K**	Stock	3.4	3.5^{e}	0.0	0.490	0.138	0.001
	Journey	3.5^{A}	$3.2^{B,f}$	0.1			
**Ca**	Stock	1.5	1.5	0.0	0.339	0.068	0.383
	Journey	1.5	1.6	0.0			
**Glucose**	Stock	4.8	5.1	0.2	<0.001	<0.001	<0.001
	Journey	4.8	7.1	0.2			
**Lactate**	Stock	0.7	0.7^{E}	0.1	0.000	0.724	0.026
	Journey	0.8	0.9^{F}	0.1			
**Cortisol**	Stock	87.1^{A}	$156.6^{B,e}$	9.5	0.003	<0.001	0.446
	Journey	68.7^{A}	126.6^{Bf}	6.8			

Table S3: Wald test P-values generated from univariate regression analysis

Predictive Variable	Clinical ESGD
Cortisol	0.076
Head Surveying	0.198
Head Tossing	0.075
Hear Rate	0.021
Interaction	0.081
Leaning	0.129
Licking	0.136
Loss of balance	0.036
Scratching	0.083
RT	0.023
Touching the cord	0.201
Turning the head	0.105
Total stress	0.070
Total balance	0.140
Total behaviour	0.074
